# Citicoline Treatment Improves Measures of Impulsivity and Task Performance in Chronic Marijuana Smokers: A Pilot BOLD fMRI Study

**Published:** 2015-09-30

**Authors:** Staci A. Gruber, Kelly A. Sagar, Mary Kathryn Dahlgren, Atilla Gonenç, Nina A. Conn, Jeffrey P. Winer, David Penetar, Scott E. Lukas

**Affiliations:** 1 Cognitive and Clinical Neuroimaging Core, McLean Hospital, Belmont, MA, USA; 2 Department of Psychiatry, Harvard Medical School, Boston, MA, USA; 3 Behavioral Pharmacology Research Laboratory, McLean Hospital, Belmont, MA, USA

**Keywords:** Citicoline, Marijuana, fMRI, Executive function, Impulsivity, Stroop Color Word Test

## Abstract

**Objective:**

Citicoline is an endogenous nucleotide that has historically been used to treat stroke, traumatic brain injury, and cognitive dysfunction. Research has also shown that citicoline treatment is associated with improved cognitive performance in substance-abusing populations. We hypothesized that marijuana (MJ) smokers who received citicoline would demonstrate improvement in cognitive performance as well as increased neural efficiency during tasks of cognitive control relative to those who received placebo.

**Method:**

The current study tested this hypothesis by examining the effects of citicoline in treatment-seeking chronic MJ smokers. In an 8-week double-blind, placebo-controlled study, 19 MJ smokers were randomly assigned via a double-blind procedure to the citicoline (8 Males, 2 Females) or placebo group (9 Males, 0 Females). All participants completed fMRI scanning at baseline and after 8 weeks of treatment during two cognitive measures of inhibitory processing, the Multi Source Interference Test (MSIT) and Stroop Color Word Test, and also completed the Barratt Impulsiveness Scale (BIS-11), a self-report measure of impulsivity.

**Results:**

Following the 8 week trial, MJ smokers treated with citicoline demonstrated significantly lower levels of behavioral impulsivity, improved task accuracy on both the MSIT and Stroop tasks, and exhibited significantly different patterns of brain activation relative to baseline levels and relative to those who received placebo.

**Conclusions:**

Findings suggest that citicoline may facilitate the treatment of MJ use disorders by improving the cognitive skills necessary to fully engage in comprehensive treatment programs.

## Introduction

Marijuana (MJ) remains the most widely used illicit drug in the United States. An estimated 18.9 million Americans aged 12 and older report MJ use in the past month, which is equivalent to 7.3% of the US population [1]. In 2012, Colorado and Washington became the first states to legalize recreational MJ and currently, 23 states plus the District of Columbia have legalized medical marijuana. As debates regarding the legalization of MJ top the nation’s headlines, the benefits of medical MJ are often emphasized. This has likely contributed to the decrease in perceived harm of MJ use, which is now approaching an all-time low. Historically, perception of risk and harm is inversely correlated with rates of use, and it is therefore not surprising that rates of MJ use increased more than 5% between 2007 and 2012 while use of alcohol, tobacco, and illicit drugs remained stable or declined over the past decade [1]. The decrease in perception of harm and accompanying increase in use are occurring despite reports of the potential adverse effects of marijuana on the brain. Contrary to popular belief, recreational marijuana use has been reported to be associated with a myriad of cognitive impairments [2–4] and alterations in brain structure [5–10] and function [11–14].

More specifically, investigations have reported reductions in executive function, specifically behavioral response inhibition in individuals who abuse marijuana [2,11,15–17], making this a critical area of investigation. For example [16], Pope and Yurgelun-Todd (1996) reported lower performance scores on tests designed to measure frontal/executive function in marijuana smokers relative to control participants, and Solowij and colleagues (2002) [17] reported significantly worse performance on a battery of measures that included attention, memory and executive function in heavy marijuana smokers relative to both lighter smokers and non-smoking controls. More recently, Lisdahl and Price (2012) [4] reported that amongst teens and emerging adults, MJ use was associated with a range of cognitive deficits, including poorer psychomotor speed, sustained attention, and cognitive inhibition. Results from previous work in our lab also suggest that MJ smokers exhibit poorer cognitive function compared to healthy, non-MJ-smoking controls, particularly on measures of executive function. Further, those who initiated regular marijuana use prior to age 16 demonstrated increased impairment relative to individuals with later onset (age 16 or later) [4]. Research conducted by Fontes and colleagues (2011) [18] yielded similar results; early onset MJ smokers (chronic use before age 15) exhibited significantly poorer performance on a variety of cognitive measures, including executive functioning, as well as sustained attention and impulse control. In examining measures of impulsivity in adolescents, MJ-smoking adolescents showed greater impairment relatively to both alcohol-using and non-substance-using adolescents, suggesting that exposure to MJ during adolescence is associated with impulsive decision-making.

Neuroimaging studies have also highlighted functional alterations in MJ smokers. A neuroimaging study by Gruber and Yurgelun-Todd (2005) [15] revealed processing deficits during frontally mediated cognitive tasks in adult MJ smokers, which resulted in altered decision-making and behavioral inhibition when compared to non-smoking controls. Using a go/no-go task during fMRI, Hester and colleagues (2009) [12] found reduced inhibitory response in current MJ smokers, who displayed a significant deficit in awareness of commission errors relative to control participants. In another fMRI study utilizing a go/no-go task, MJ smokers demonstrated increased activation, despite similar task performance, further suggesting inhibitory processing alterations in MJ users [14]. A more recent study conducted in our lab [11] also revealed altered patterns of activation in chronic MJ smokers relative to healthy controls during the performance of an inhibitory task. These findings highlight the likelihood that chronic adult MJ smokers have altered frontal system function, and suggest that they may have cognitive deficits that compromise overall function. The impact of MJ use on brain structure and function raises an important public health issue and is of great concern, as rates of MJ use have risen in recent years, especially among teens [19].

Citicoline (CDP-choline; cytidine 5′-diphosphocholine) is an endogenous nucleotide that plays an important role in cellular metabolism as a precursor to phosphotidylcholine [20]. It has a number of putative mechanisms of action, including as a phospholipid precursor for neuronal membrane repair, for prevention of β-amyloid deposition, and for promotion of neurotransmitter systems [21]. Citicoline has been used therapeutically for stroke, traumatic brain injury, and cognitive dysfunction in the elderly [21,22], has been reported to be beneficial in slow advancing neurodegenerative disorders [23] and is currently marketed as a medication in Europe and Japan and as an over-the-counter dietary supplement in the United States. Composed of choline and cytidine, citicoline is a reputed precursor of acetylcholine (Ach), and thought to promote synthesis and transmission of neurotransmitters that are important for memory and attentional focus. One possible mechanism for the role of citicoline as a nootropic is to increase the availability of ACh, which might facilitate the enhancement of attention, even in healthy adults [24]; beneficial effects of citicoline on memory have been observed in a number of studies of healthy older adults. Agnoli and colleagues conducted a double-blind, placebo-controlled study of older adult participants with mild to moderate memory problems and administered 1,000 mg/day of citicoline for 4 weeks and tested them using the Randt Memory Test. Only the high-IQ individuals demonstrated improved acquisition efficiency, with no differences between drug and placebo groups on composite measures of encoding, organization or cognitive efficiency [25]. Spiers et al. (1996) [26] also conducted a 3-month, double-blind, placebo-controlled study of citicoline (1,000 mg/day) and found no effects on verbal memory. However, in a crossover study of a subgroup of individuals with relatively poor memory, administration of a higher dose of citicoline (2,000 mg/day) for 2 months significantly improved scores on the Logical Memory subtest of the Wechsler Memory Scale [26]. Alvarez and colleagues (1997) [27] administered either 500 or 1,000 mg/day of citicoline or 300 mg/day of citicoline + nimodipine, a calcium channel blocker, to memory-impaired older adults for 4 weeks and reported that citicoline improved memory in free recall (word and object) tasks, but not recognition tests. A review paper highlighted that, in general, individuals with poor or inefficient memories benefit from citicoline treatment, demonstrating improvements in memory efficiency, acquisition efficiency, delayed recall, and logical memory [21]. These cognitive faculties are fundamental to global functioning and invaluable assets to have when engaged in treatment services.

Citicoline has also been demonstrated to improve cognitive function in substance abusers. In a study designed to examine safety and efficacy of citicoline in reducing drug related craving, Renshaw et al. (1999) [28] examined a sample of cocaine-dependent individuals who were randomized to receive 14 days of treatment with either 500 mg, bid of citicoline or placebo. Citicoline decreased self-reported cocaine craving relative to placebo, and no individuals reported any side effects. In addition, placebo-treated participants had twice as many marijuana-positive urine screens than the citicoline-treated participants, suggesting that citicoline may affect drug-taking behavior in general, and not be specific for any single drug class. In order to examine the safety of citicoline and potential cardiovascular effects, Lukas et al. (2001) [29] administered cocaine challenges to cocaine-dependent individuals who had been pretreated with citicoline or placebo. The participants pretreated with citicoline had no potentiated cardiovascular, physiologic or subjective effects after cocaine, suggesting that citicoline is safe to use in this population. Brown and colleagues (2007) [30] conducted a 12-week randomized placebo controlled trial of citicoline in patients with both bipolar disorder and cocaine dependence and examined a number of clinical, cognitive and substance use variables. Results indicated a significant effect for the citicoline treated group on the alternative word list of the Rey Auditory Learning Test (RAVLT), a measure of verbal learning. Further, citicoline-treated individuals had a significantly lower probability of having cocaine-positive urine at the end of the study; placebo-treated patients were 6.4 times more likely to have used cocaine than those who received citicoline. Finally, in a study by Yoon et al. (2010) [31], investigators examined the effect of 2,000 mg/day of oral citicoline or placebo on methamphetamine-dependent individuals using magnetic resonance spectroscopy (MRS) methods in frontal brain regions. Levels of N-acetyl-aspartate (NAA), considered a measure of neuronal integrity, were significantly higher in the citicoline-treated individuals as compared to those who received placebo. Further, changes in NAA levels were *positively* associated with total number of negative urine results, indicating lower drug use in citicoline-treated participants. The authors suggest that citicoline treatment may yield direct or indirect neuroprotective effects and that further studies are warranted to explore the long-term efficacy of citicoline for abstinence. Taken together, results from studies of healthy adults and substance abusers suggest that treatment with citicoline may improve cognitive function and impact neural processes. Based on these findings, we hypothesized that in chronic, heavy marijuana smokers, eight weeks of treatment with oral citicoline would result in improved cognitive performance and increased neural efficiency during tasks of cognitive control relative to individuals who received placebo.

## Method

### Participants

In a double-blind clinical trial (clinicaltrials.gov: NCT00158249) of oral citicoline, we examined data from nineteen chronic MJ smokers (of the 43 individuals who provided informed consent, 30 met eligibility criteria, and 19 completed the study). These participants, who responded to advertisements placed in the Greater Boston community for treatment-seeking marijuana users, were randomized to receive either 2,000 mg of citicoline (*n* = 10) or placebo daily (*n* = 9). Prior to participation, study procedures were explained, and participants were required to read and sign an informed consent form approved by the McLean Hospital Institutional Review Board, which described the procedures and voluntary nature of the study. All participants received the Structured Clinical Interview for DSM-IV, Patient Edition (SCID-P; First et al., 1994) to assess for Axis I pathology as well as a physical examination which included a medical history and physical exam, blood draw, urinalysis, and electrocardiogram to ensure good physical health prior to participation. To qualify for study entry, all participants had to be between the ages of 18–45 without a history of major head trauma, cardiac problems, or other physical health problems and had to meet DSM-IV criteria for MJ abuse or dependence. Once enrolled, participants completed functional magnetic resonance imaging (fMRI) during the completion of two tasks of inhibitory function previously used with success [11,32] at baseline and after 8 weeks of treatment. All participants also completed daily MJ use diaries, previously utilized with substance-using populations in the past [33]. These diaries are designed for participants to document frequency (smokes/day) and magnitude (grams/day) of MJ use, as well as the total dollar estimate of the amount of MJ used and level of MJ craving. Each day, craving for MJ over the previous 24-hour period was assessed using a 10-point Likert scale, ranging from “none at all” to “extremely high.” Further, participants were required to abstain from MJ use for at least 12 hours prior to scanning sessions to ensure that they were not acutely intoxicated at the time of testing, and were told that they would be tested upon arrival. This method has been used with success in previous studies [3,9,11]. All participants also completed clinical rating scales during study visits, including the Barratt Impulsiveness Scale [34], a robust 30-item, self-report scale that provides reliable measures of impulsivity in multiple domains.

### Study Design

All participants completed the Multi Source Interference Test (MSIT) and the Stroop Color Word Test while undergoing fMRI, as described previously [11,32,35]. Briefly, during the MSIT, they were presented with sets of three numbers, containing combinations of 0, 1, 2, and 3, for 1.75 seconds with a prerelease of 0.5 seconds, for a stimulus presentation of 1.25 seconds and an interstimulus interval of 0.5 seconds, yielding a total run time of 6 minutes and 36 seconds. In each set, one number was always different from the other two (distractor) numbers, and participants were instructed to report the identity of the number that differed from the distractors using a fiber optic button box. During control trials, distractor numbers were always zeros and the target number was always presented in a matching position to the corresponding button on the button box (i.e., 100, 020, 003). During the interference condition, distractors were numbers other than zero and the position of the target number was never the same as its identity (i.e., 211, 232, 331, etc.). The entire task was comprised of four blocks of control trials alternating with four blocks of interference trials. Each block consisted of 24 presentations of number sets with a total of 192 number sets presented.

The Stroop task, previously used by the authors [32], also utilized a block design, and each of three scanning epochs was divided into five segments. During the odd numbered segments, participants were asked to look at a fixation point on the screen, whereas during even numbered segments, participants were asked to perform one of the three subtests. Each individual performed the following three tasks: (1) Color Naming: they were asked to report the color of randomly sequenced color stimuli, represented as color blocks, which were printed in red, blue and green ink; (2) Word Reading: participants were asked to read color words (“red,” “blue,” and “green”) printed in black ink, which established a response set to reading color words; and (3) Interference: participants were presented words which were printed in an incongruent ink color (i.e., “red” printed in blue ink, “green” printed in red ink). During this subtest, participants were asked to report the ink color; to succeed, one must suppress the automatic, over learned tendency to read the color word. Given the importance of establishing a response set for color naming and word reading, the three conditions were always administered to study participants in the order described.

All stimuli were generated from a laptop computer running E-Prime software and presented via a high resolution, rear-projection system onto a translucent screen located at the rear of the scanner (Resonance Technology, Inc.) and viewed through a mirror mounted on the head coil. Participants completed a practice session for each task in order to familiarize them with the tasks and button box. Performance on each task was quantified by: 1) percent accuracy on each of the task conditions; 2) reaction time for each response (for the MSIT only; Stroop stimuli appeared as a series of six stimuli on the screen at once); and 3) errors of omission (no response given) and commission (incorrect response) per task condition.

### Imaging methods

Imaging was performed on a Siemens TIM Trio whole body 3T MRI scanner (Siemens Corporation, Erlangen, Germany) using a 12-channel phased array head coil. During the cognitive challenge tasks, 40 contiguous coronal slices were acquired from each participant, providing whole brain coverage (5 mm thick) and images were collected every 3 seconds using a single shot, gradient pulse echo sequence (TR = 3000 ms; TE = 30 ms, flip angle = 90°, with a 20 cm field of view and a 64 × 64 acquisition matrix; in plane resolution 3.125 × 3.125 × 3.125 mm). For the MSIT, a total of 132 images per slice were collected, while for the Stroop task, a total of 50 images per slice were collected in order to ensure comparability of tasks with previous studies [11,32].

### Image processing and analysis

fMRI images were analyzed using SPM8 (Wellcome Trust Center for Neuroimaging, UK). Blood oxygen level dependent (BOLD) fMRI data were corrected for motion, normalized to Montreal Neurological Institute (MNI) stereotactic space, and spatially smoothed using an isotropic Gaussian kernel 6 mm full width at half maximum (FWHM). Statistical parametric images were calculated individually for each participant and each task, using a general linear model that accounted for task-related changes, with each condition modeled as a block design with a boxcar waveform. At the first level, three regressors were fit to the data, including the baseline fixation condition, the control condition, and the interference condition. Both the control and interference conditions of the MSIT included four active blocks each comprised of 42 s stimulation periods. For the Stroop, each scan epoch consisted of 4 on/off cycles with a 30 s fixation period prior to the presentation of any stimuli. Activation was averaged across these blocks, and no attempt was made to adjust for individual item performances. These contrast images were subsequently entered into a second level model, subjected to voxel-wise t-tests to assess statistical significance using 1-sample t-tests. The citicoline and placebo groups were compared using between group t-tests and the primary contrasts examined were the difference between the interference condition and the control condition of each task (i.e. MSIT Interference – MSIT Control; Stroop Interference – Stroop Color Naming). As previous work from our group has demonstrated specific differences within the anterior cingulate cortex (ACC), we applied an ACC region of interest (ROI) mask using the Wake Forest University Pickatlas utility to restrict analyses to this area. Voxel-wise comparisons restricted to this ROI were evaluated at *p* < 0.005 (uncorrected), *k* ≥ 10 contiguous voxels. Further, only clusters that exceeded a false discovery rate (FDR) correction of *p* < 0.05 are included.

### Statistical analyses

Independent sample *t* tests were used to assess between-group differences at baseline and at the end of treatment (week 8) when the assumptions for parametric tests were met; otherwise, non-parametric Mann-Whitney U tests were performed. To determine treatment group differences in MJ use over the course of the study from baseline to the end of treatment (week 8), 2×2 mixed model (treatment × time) analyses of variance (ANOVAs) were used for complete cases. Independent sample *t* tests on the difference scores (Baseline Score – Treatment Week 8 Score) were used to assess performance change over time for the neurocognitive tasks. Additionally, in order to account for baseline levels of dependent variables (e.g., BIS scores, Stroop and MSIT percent accuracy), percent change scores were also calculated and compared using independent sample *t* tests. Percent change scores for Stroop and MSIT percent accuracy were calculated using the following formula: ((Treatment week – Baseline Score/Baseline Score) * 100) where higher scores after treatment reflect improvement. However, because lower BIS scores over time indicate improvement, a slightly modified formula was utilized to more accurately reflect the data: ((Treatment week – Baseline Score/Baseline Score) * −100).

## Results

### Participant demographics

As reported in Table 1, independent samples *t* tests (2-tailed) confirmed that participants in both groups were well matched for age, education, and IQ, as measured by the Wechsler Abbreviated Scale of Intelligence (WASI; Wechsler, 1999) [36]. Additionally, due to violations of the normality assumption, non-parametric, Mann-Whitney U tests (2-tailed) were used to compare baseline measures of marijuana use (age of onset of regular marijuana use, duration of marijuana use (yrs), as well as daily, weekly, monthly, and lifetime marijuana uses) and on measures of alcohol and nicotine use (number of alcoholic drinks consumed per week and number of cigarettes smoked per day). These analyses indicated that both groups were well matched for marijuana, alcohol, and nicotine use at baseline.

### Marijuana use

The 2×2 mixed model (Treatment × Time) ANOVA analyses revealed a main effect of Time. According to participants’ diary entries detailing their daily MJ use, both groups reported significant decreases in MJ use between baseline and 8 weeks of treatment. More specifically, craving (*F*(1,16) = 3.61, *p* = .038, 1-tailed), frequency of use (*F*(1,16) = 4.87, *p* = .021, 1-tailed), grams consumed (*F*(1,16) = 6.17 *p* = .012, 1-tailed), and dollar amount estimate of MJ used (*F*(1,16) = 6.17 *p* = .012, 1-tailed) all significantly decreased between baseline and 8 weeks of citicoline treatment or placebo. However, results demonstrated that these effects were not significantly influenced by the treatment condition (citicoline vs. placebo); no main effect of Treatment or Time × Treatment interaction effect was discovered for treatment condition on craving, daily use (frequency and grams used), or amount spent on MJ.

### Behavioral assessment: Impulsivity

**Figure 1:**
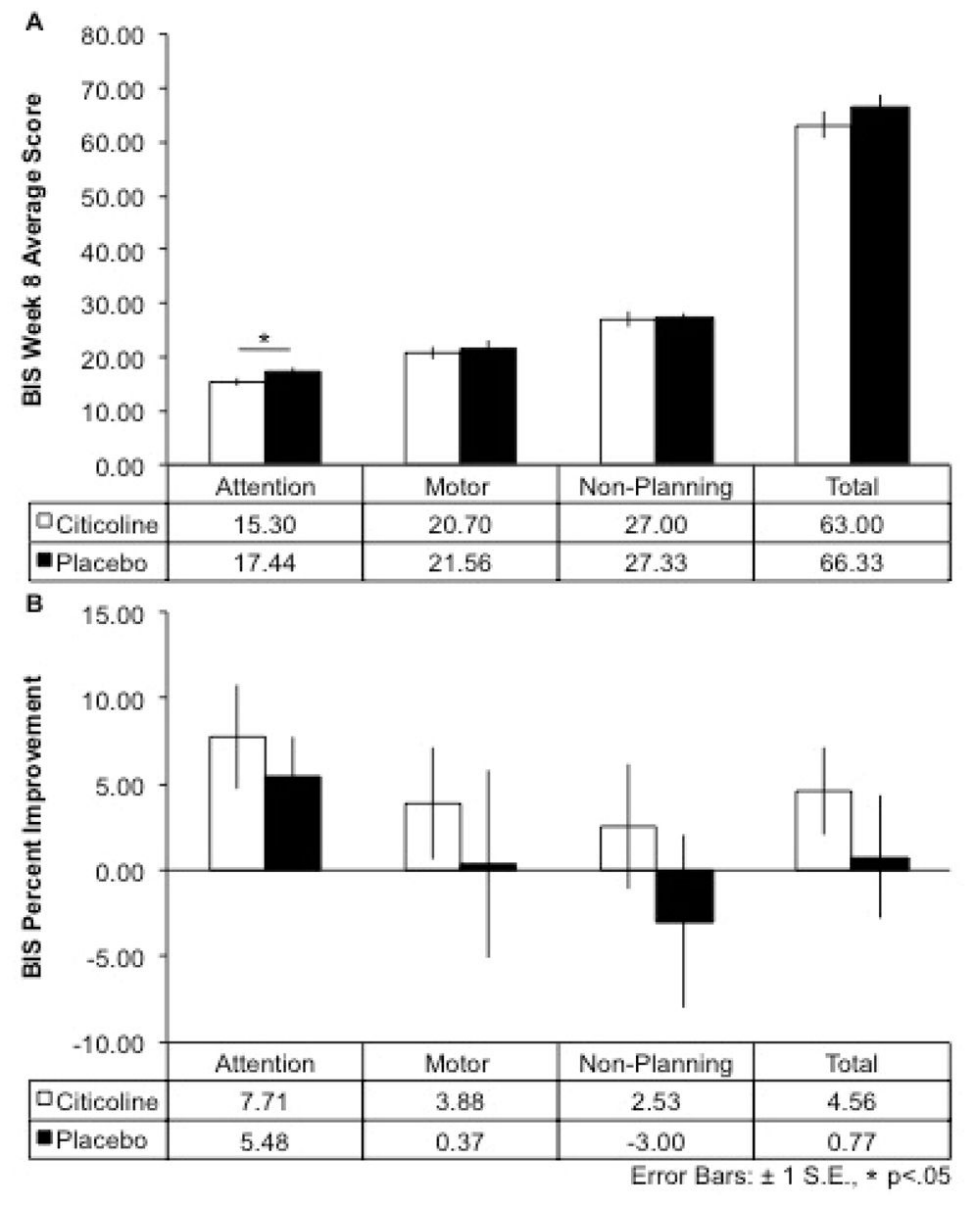
Barratt Impulsiveness Scale (BIS) Scores and Percent Improvement After 8-Week Citicoline Treatment A.) After 8 weeks of treatment, the citicoline-treated group showed a significant reduction in attentional impulsivity compared to the placebo-treated group (*t*(17) = 2.20, *p* = .02, 1-tailed). B.) The citicoline-treated group showed greater percent improvement from baseline to week 8 for all BIS subscales as well as total BIS scores. *Note: since lower scores are indicative of reduced impulsivity, percent improvement has been reciprocally plotted to aid visualization. Positive values indicate greater reduction of impulsivity.*

Impulsivity was assessed with the Barratt Impulsiveness Scale (BIS), which includes self-report measures of attention, motor, non-planning, and total impulsivity subscales. Despite no significant between-group differences in impulsivity at baseline, following the 8-week treatment period, the citicoline-treated group had significantly lower scores on the attention subscale of the BIS compared to the placebo group (15.30 ± 2.11 vs. 17.44 ± 2.13; *t*(17) = 2.20, *p* = 0.02, 1-tailed; Figure 1A). The citicoline-treated group demonstrated greater percent change (i.e., lower scores) in all BIS subscores as well as total BIS scores compared to the placebo group over the 8-week treatment period, although this did not reach statistical significance (Figure 1B).

### Neurocognitive performance

**Figure 2:**
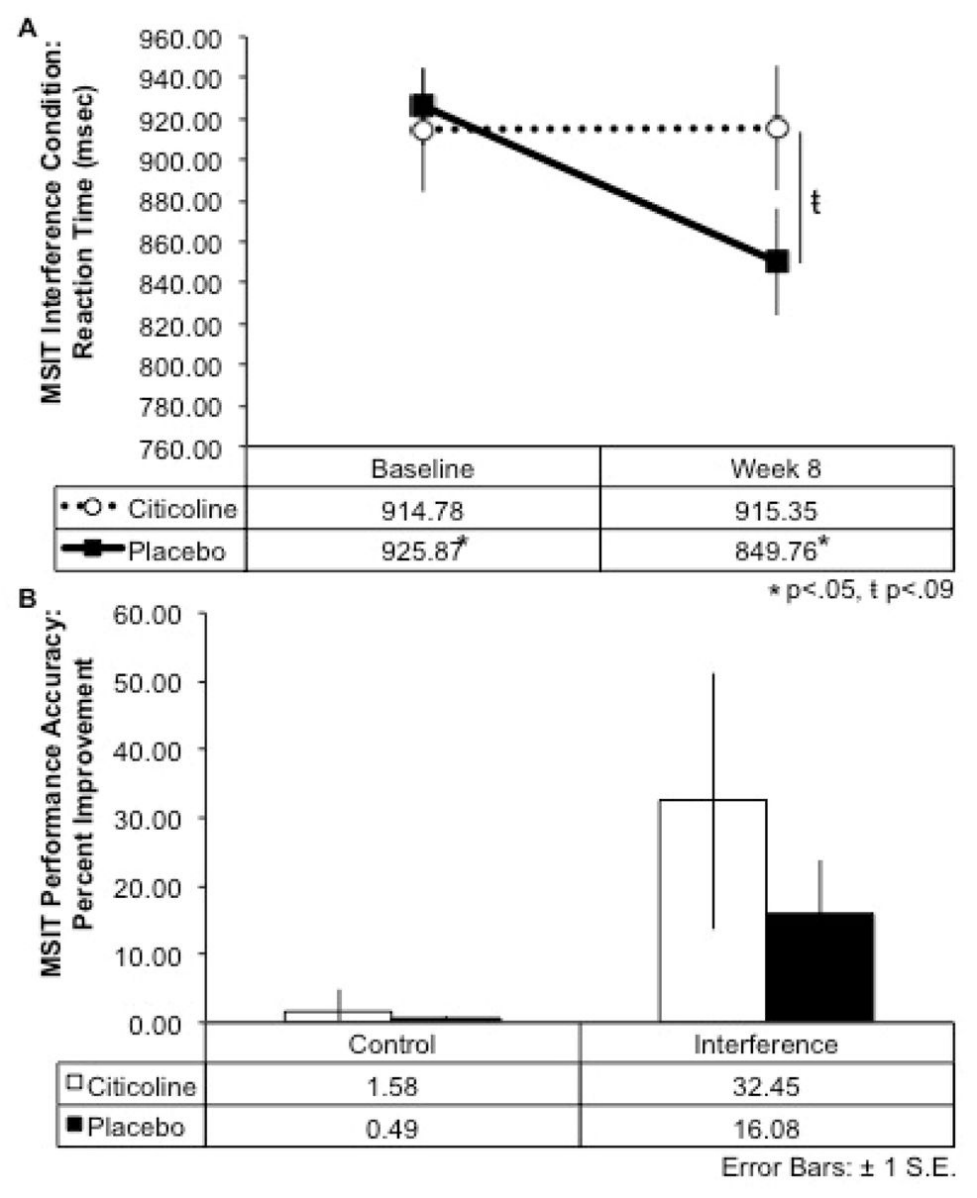
Multi Source Interference Test (MSIT) Percent Improvement of Performance Accuracy and Reaction Time After 8-Week Citicoline Treatment A.) At baseline, the citicoline and placebo-treated groups exhibited similar reaction time during the interference condition of the MSIT; however, by week 8 the citicoline-treated group exhibited a trend for slower reaction times than the placebo-treated group. Further, the citicoline-treated group generally performed the task more slowly at week 8 than at baseline, while the placebo-treated group performed the task significantly faster over time (*t*(17) = 2.32, *p* = .02, 1-tailed). B.) The citicoline-treated group showed greater percent improvement from baseline to week 8 during both the control and interference conditions of the MSIT.

**Figure 3:**
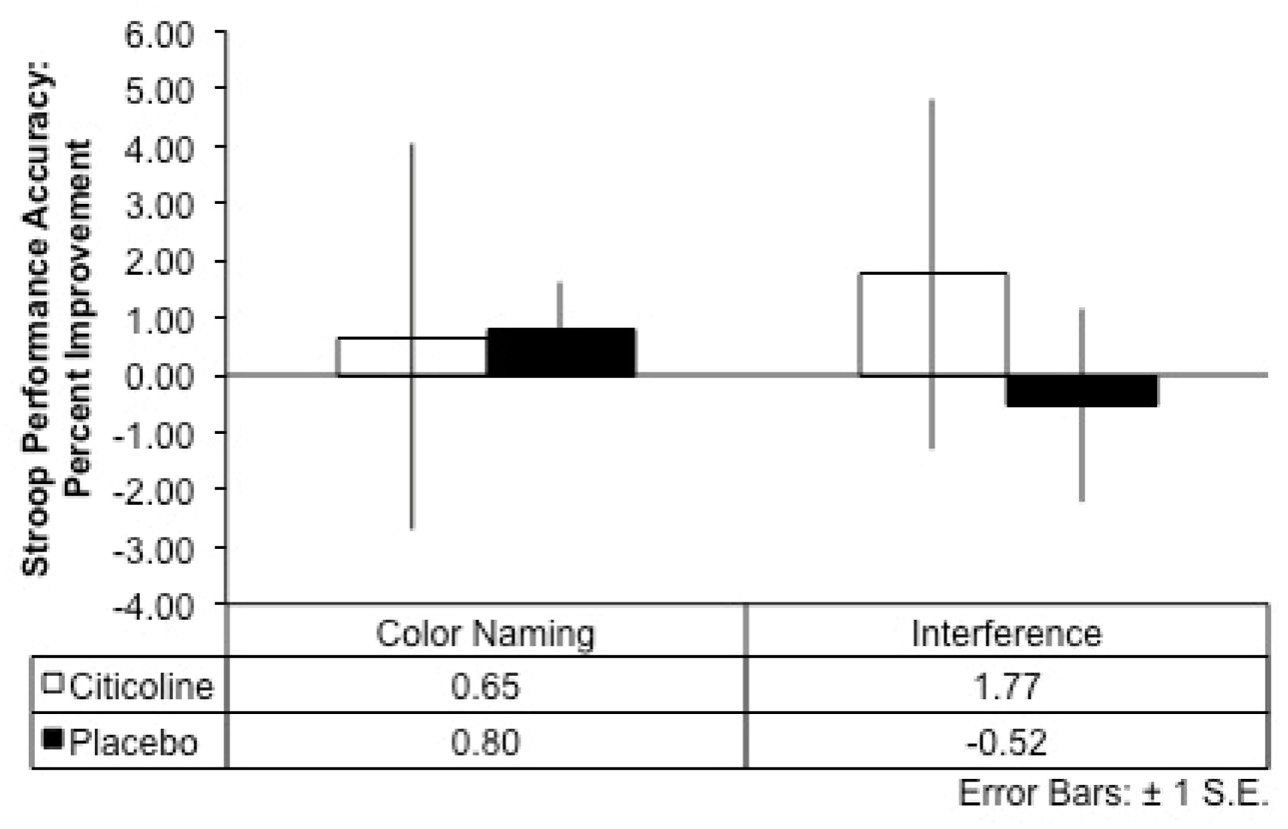
Stroop Color Word Test: Performance Accuracy after 8-Weeks of Citicoline Treatment The citicoline-treated group showed greater percent improvement relative to the placebo-treated group during the Interference condition of the Stroop between baseline and 8 weeks of treatment.

For the interference condition of the MSIT, results indicated that over the course of the eight-week treatment period (difference scores: Baseline Score – Treatment Week 8 Score), the citicoline-treated group exhibited a *smaller* change in reaction time from baseline to week 8 and performed the interference condition of the task slightly more slowly over time, while the placebo group demonstrated a larger magnitude of change in reaction time from baseline, and exhibited faster reaction times over the course of treatment (*t*(17) = 2.32, *p* = .02, 1-tailed; see Figure 2A). Further, the citicoline-treated group demonstrated greater percent improvement in MSIT interference task accuracy relative to the placebo-treated group following 8 weeks of treatment, although this did not reach statistical significance (Figure 2B). In addition, during the completion of the interference condition of the Stroop Color-Word Test, the citicoline-treated group also demonstrated greater percent improvement on performance accuracy relative to the placebo-treated group; however, this result did not reach statistical significance (Figure 3).

### Functional Magnetic Resonance Imaging (fMRI) results

In order to assess the impact of citicoline treatment on brain activation patterns, we analyzed data from both baseline and the week 8 scans and completed contrast analyses for the anterior cingulate cortex (ACC), a critical region for inhibitory processing. For the MSIT (Interference task minus Control task), the citicoline-treated group demonstrated a shift from posterior to more anterior ACC activation, with a corresponding slight increase in total voxel cluster size from baseline to treatment week 8 (Figure 3, Table 2) In contrast, while the placebo-treated group also demonstrated activation in posterior ACC at baseline, following 8 weeks of treatment activation shifted slightly more anteriorly and remained in posterior and mid ACC (Figure 3, Table 2).

**Figure 4:**
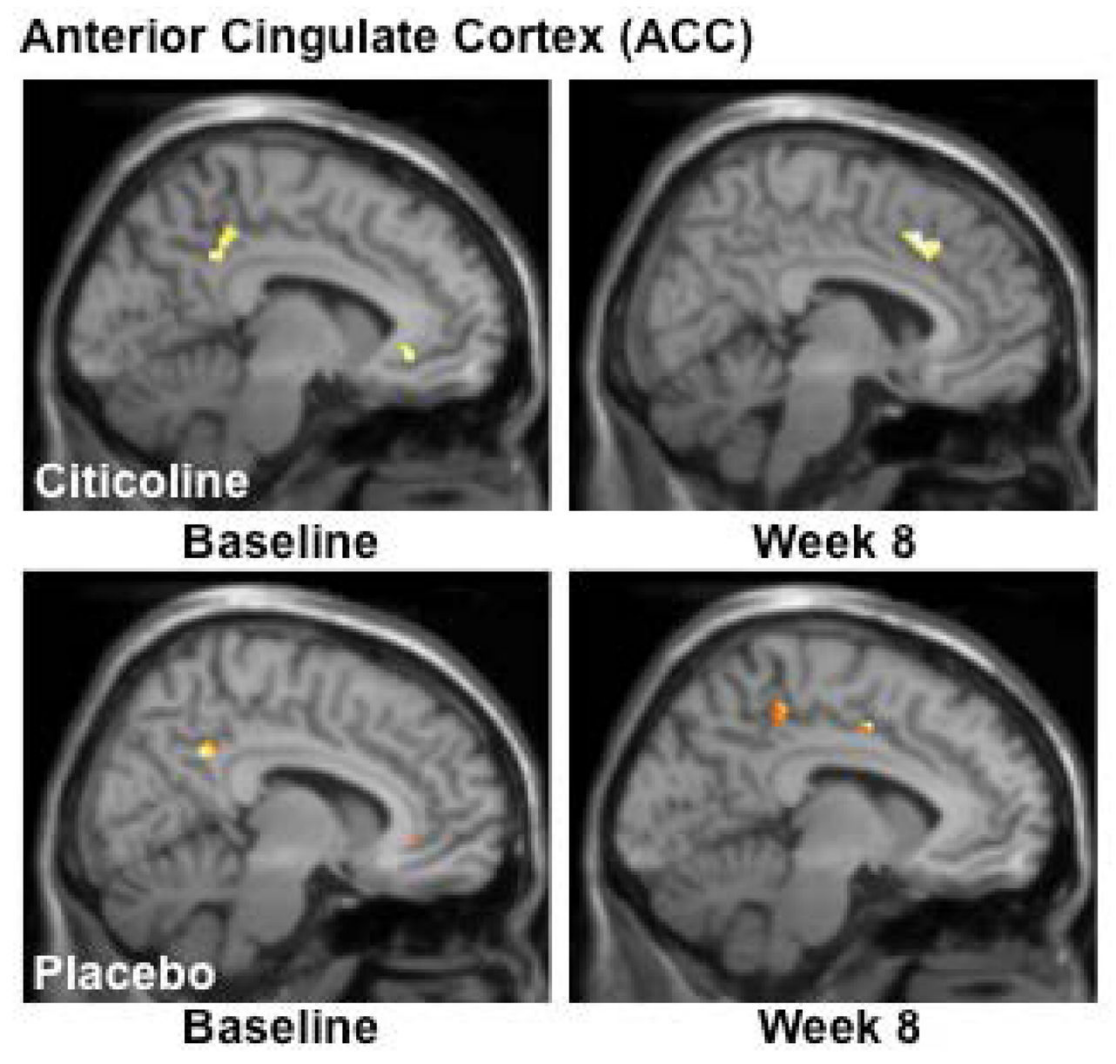
fMRI Analysis of the Multi-Source Interference Task (MSIT): Interference-Control Condition Analyses of MSIT (Interference task minus Control task) activation revealed a shift from posterior to more anterior ACC activation, with a corresponding slight increase in total voxel cluster size from baseline to treatment week 8 in the citicoline-treated group. The placebo-treated group also demonstrated activation in posterior ACC at baseline, but following 8 weeks of treatment, activation shifted slightly more anteriorly and remained in posterior and mid ACC. (k = total cluster size with p < 0.01(uncorrected) and a threshold of ≥ 10 voxels).

**Figure 5:**
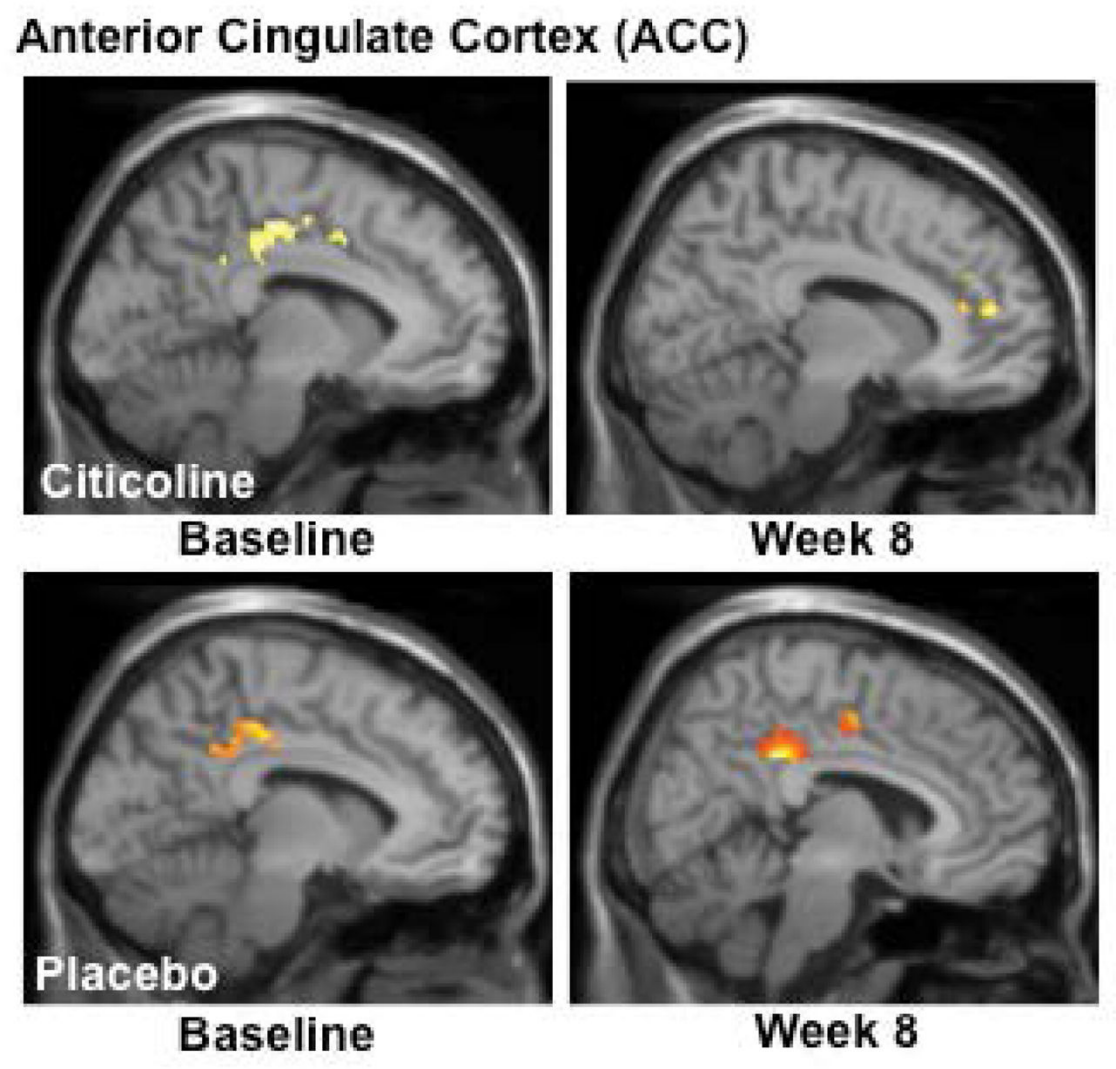
fMRI Analysis of the Stroop Color Word Task: Interference – Color Naming Activation during Stroop (Interference condition minus the Color Naming condition) highlighted a shift from posterior/midcingulate to genual cingulate activation and a corresponding reduction in total voxel cluster size in the citicoline-treated group. The placebo-treated group demonstrated a pattern of posterior ACC activation at baseline which persisted at the 8 week scan, with only minor midcingulate activation and an increase in total voxel cluster size. (k=total cluster size with p < 0.01(uncorrected) and a threshold of ≥ 10 voxels).

Results from the interference minus color naming contrast of the Stroop Color Word test revealed that the citicoline treated group demonstrated a shift from posterior/midcingulate to genual cingulate activation with a corresponding reduction in total voxel cluster size following 8 weeks of treatment (see Figure 4, Table 3). The placebo-treated group demonstrated a pattern of posterior ACC activation at baseline which persisted at the 8 week scan, with only minor midcingulate activation. This pattern also accompanied an increase in total voxel cluster size (see Figure 5, Table 3); however, no activation in genual or near genual regions was noted for the placebo group following the treatment period.

## Discussion

As hypothesized, chronic MJ smokers randomized to receive 2,000 mg of citicoline per day demonstrated significantly *lower* levels of behavioral impulsivity, *improved* task accuracy on two measures of inhibitory function, and exhibited a significantly *different* pattern of brain activation patterns relative to those who received placebo. While the citicoline-treated group demonstrated significantly different patterns of activation during both inhibitory tasks following 8 weeks of treatment, the placebo-treated group remained largely unchanged. Taken together, these data demonstrate that administration of citicoline may reduce behavioral impulsivity, improve cognitive performance, specifically on measures of inhibitory control, and improve neural processing in chronic, heavy MJ smokers after only a relatively brief treatment period.

These data are largely consistent with previous investigations, including work from our group. In earlier studies, we demonstrated that short-term citicoline treatment can have a beneficial effect on cocaine- and marijuana-use behaviors [28,29,33] and that it also positively impacts sleep patterns [37]. These studies were initially undertaken as citicoline had been used as a neuroprotective agent in individuals with a variety of neurological diosorders such as stroke, dementia and Parkinson’s Disease [20,38–40]. The unique pharmacology of citicoline suggested that it may be useful as a pharmacotherapy for individuals with substance abuse via two mechanisms: repair of biological membranes and increased dopamine levels. There is also ample evidence that chronic drug abuse results in abnormalities in cerebral blood flow. In particular, an early study using single photon emission computed tomography (SPECT) revealed that chronic cocaine and opiate abusers have significant perfusion deficits [41]. These deficits appear similar to those seen after a stroke or head injury, one reason that substantiates the application of citicoline in this manner.

Increased impulsivity has been labeled a metric of loss of executive control, and a recent review noted the value of neuroimaging studies in assessing its relationship to MJ use [42]. Further, previous investigations have reported higher levels of impulsivity in MJ smokers relative to non-MJ smoking control individuals [8,9,43]. The significant reduction in self-reported impulsivity observed in the citicoline-treated group underscores the likelihood that the improved task accuracy may, at least in part, be moderated by slower, less impulsive responses. Taken together, these findings demonstrate that citicoline may improve the MJ-related impairment in cognitive performance, specifically on measures related to impulsivity and inhibitory function. The significance of this finding is that reducing behavioral implusivity is a critical component in continued abstinence from drugs of abuse including tobacco, ecstasy and MJ [44–46]. While there are no approved or universally accepted treatments for MJ use disorders, a number of likley candidate medication and behavioral approaches have been posited. Data from the present study suggest that regardless of the therapeutic approach taken, citicoline may be a useful adjunct treatment for MJ use disorders.

Further, studies have reported that some individuals, including adolescents, who attempt to quit smoking marijuana experience withdrawal symptoms [47,48], which likely contributes to relapse [49]. As CNS neuronal phospholipid levels are significantly depleted in chronic substance abusers, we hypothesize that replenishment of these important compounds may help restore homeostasis or possibly repair the resultant neurological/neurochemical deficits that result from chronic substance abuse. In fact, citicoline has been shown to increase phospholipid levels, underscoring the likelihood that individuals with substance abuse may benefit from citicoline treatment.

To date, treatment strategies for drug abuse have often focused on specific receptors and/or neurotransmitters, and there are currently no FDA- approved medications that are useful in treating MJ-related disorders. While the present data do not support the use of citicoline as a first line medication that will reduce abuse on its own, it provides an alternative interpretation of its likely utility. The fact that some of the more promising treatments for drug and alcohol abuse include behavioral strategies [50] is a key development in a transition away from pure pharmacologic approaches. In order for therapeutic treatments like Cognitive Behavioral Therapy (CBT), contingency management, mindfulness, etc. to be maximally effective, patients must be able to accurately encode the information provided to them, plan and decide when to use specific behavioral strategies, maintain cognitive set during the execution of the strategies, and utilize feedback regarding their behavior, each of which requires executive function. As citicoline has been shown to improve cognitive function, specifically in the executive function domain, treatment with citicoline may facilitate an individual’s ability to take full advantage of the behavioral therapy. This may provide a basis for citicoline to serve as an adjunct to a more comprehensive treatment plan that incorporates all of the elements of behavioral and pharmacological approaches to treating the addictions.

Interestingly, study results revealed that participants across both treatment groups (citicoline and placebo) reported decreases in MJ use patterns (frequency, grams of MJ consumed, and amount spent on MJ) as well as MJ-related cravings over the course of the study. This finding may be related to the potential ‘insight’ that particicpants often gain during the course of a clinical trial. Specifically, the decreases noted in MJ related patterns across all study participants may suggest that for at least some individuals, closely monitoring their own MJ use may positively impact the ability to decrease MJ smoking. Therefore, daily MJ use diairies may prove to be a low-cost, feasible method for assisting in reduction of MJ use in treatment-seeking populations.

The current investigation has several limitations, including a relatively small sample size, often the case for imaging-based studies within clinical trials. Contributing to this small sample size was also some degree of subject attrition. It is of note, however, that both the citicoline and placebo groups experienced similar attrition rates, suggesting that attrition was unrelated to citicoline use, but instead was more likely a consequence of the 8-week treatment period, which inherently required a significant time commitment from participants due to multiple weekly study visits. Future investigations should include larger study samples in order to better address issues of subject attrition and ensure generalizability of findings to other populations.

In addition, both groups demonstrated variability in their performance of the cogntive tasks, a common finding for individuals with substance use. It is of note that both groups in the current study were well-characterized with regard to cognitive function, and did not differ on any demographic variable (age, education, IQ, duration of MJ use) at baseline. This underscores the likelihood that the findings of improved cogntive performance, reduced self-reported impulsivity, and changes in patterns of brain activation following citicoline treatment are reflective of the actual treatment intervention and *not* simply the product of baseline between-group differences.

Further, a self-report method of MJ use was utilized in the current study in which participants were required to report frequency of use as well as weight (grams) used and estimated dollar amount of MJ used. It is possible that some individuals may have utilized an actual scale to report MJ weight, while others estimated or subjectively reported the amounts they believed they were smoking. Finally, all participants in the current study were asked to abstain from MJ use for a minimum of 12 hours prior to their study visit in order to ensure they were not acutely intoxicated at the time of scanning. They were also led to believe that we would be able to determine the time of their last MJ use once in the laboratory, a method we have previously used with success [3,8,9,11]. While we cannot be certain that all participants fulfilled this requirement, all reported compliance with the abstinence request and fully expected investigators would be able to tell if they had used the drug since the previous evening upon arrival at the lab. Indeed, no individual appeared intoxicated, and all were able to complete the cognitive tasks with minimal effort.

## Conclusions

Data from the present study suggest that citicoline treatment may improve cognitive performance, specifically with regard to tasks requiring executive function. Further, individuals receiving citicoline treatment demonstrated reduced self-reported levels of impulsivity and more efficient patterns of brain activation during inhibitory tasks relative to those who received placebo. These findings have significant implications for current public policy, especially given the rise in MJ use among emerging adults. The recent increase in states’ legislation to decriminalize MJ use or allow for its use in treating medical conditions may cause rates of use to rise among the general public. This pattern is reflected in the National Trends reported in the Monitoring the Future data, where perceived risk of MJ continues to fall while MJ use rises simultaneously [51]. Given this movement, the present results are even more compelling, as they underscore the potential utility of investigating alternative, adjunctive therapies for MJ use disorders, and may have a significant impact on treatment strategies.

## Figures and Tables

**Table 1:** Demographic and Behavioral Data

Variable	Citicoline Group *n* = 10	Placebo Group *n* = 9	*p* (2-tailed)
Handedness	10R, 0L	9R, 0L	-
Age	27.70 ± 6.98	30.00 ± 7.09	NS
Education (yrs)	14.10 ± 2.02	14.22 ± 2.28	NS
Full Scale IQ (WASI)^a^	114.20 ± 12.07	115.00 ± 8.99	NS
Age of MJ Onset (yrs)	16.80 ± 3.94	19.00 ± 2.50	NS
Duration of MJ Use (yrs)	10.90 ± 8.90	11.33 ± 5.66	NS
Lifetime MJ Uses	17,619.11 ± 29,157.37	14,387.11 ± 20,326.28	NS
Monthly MJ Uses	101.20 ± 123.03	103.78 ± 83.63	NS
Drinks per Week	5.53 ± 4.49	3.04 ± 2.19	NS
Tobacco Cigarettes per Day	1.70 ± 3.34	3.57 ± 6.78	NS
**MJ Use Per Day**			
Baseline Average	4.3 ± 4.2	3.3 ± 1.4	NS
Treatment Week 8 Average	3.8 ± 3.1	2.5 ± 2.0	NS

a WASI – Wechsler Abbreviated Scale of Intelligence

**Table 2:** MSIT: Local Maxima for Group Comparisons with Anterior Cingulate Cortex (ACC) Region of Interest (ROI).

*TASK CONDITION* Group/Visit Region	Cluster Size (Voxels)	x	y	z	SPM {t}	Voxel *p* Uncorrected
***MSIT Interference>MSIT Control***						
**Citicoline/Baseline**						
Left Anterior Cingulate	25	−8	32	−6	4.25	.001
Left Precuneus	135	−4	46	36	4.13	.002
**Citicoline/Week 8**						
Right Medial Frontal Gyrus, BA8	181	6	18	44	4.14	.002
**Placebo/Baseline**						
Right Precuneus	119	8	52	32	5.11	.001
Left Anterior Cingulate, BA32	81	−6	34	−4	3.00	.008
Right Paracentral Lobule, BA5	28	4	36	50	2.69	.009
**Placebo/Week 8**						
Left Cingulate Gyrus, BA24	15	−8	−2	44	13.72	>.001
Right Medial Frontal Gyrus	10	8	50	14	11.37	>.001
Left Paracentral Lobule BA5	57	−8	38	52	8.23	>.001

**Table 3:** Stroop: Local Maxima for Group Comparisons with Anterior Cingulate Cortex (ACC) Region (ROI).

*TASK CONDITION* Group/Visit Region	Cluster Size (Voxels)	x	y	z	SPM {t}	Voxel *p* Uncorrected
***Stroop Interference***						
**Citicoline/Baseline**						
Right cingulate gyrus, BA 24	140	12	2	42	11.23	<.001
Right cingulate gyrus, BA 24	60	4	24	34	9.44	<.001
Left cingulate gyrus, BA 32	14	−6	6	42	9.27	<.001
Right anterior cingulate, BA 24	26	2	34	12	9.14	<.001
Right medial frontal gyrus, BA 9	13	8	42	22	11.02	<.001
Left medial frontal gyrus, BA 6	183	−6	22	48	10.77	<.001
Left medial frontal gyrus, BA 10	14	−4	50	10	10.72	<.001
Left superior frontal gyrus, BA 9	44	14	50	32	10.48	<.001
**Citicoline/Week 8**						
Right cingulate gyrus, BA 24	19	6	10	28	8.82	<.001
Left anterior cingulate, BA 24	16	−4	28	−6	7.98	<.001
Right medial frontal gyrus, BA 9	16	8	38	28	6.94	<.001
Right medial frontal gyrus, BA 10	269	10	48	14	10.90	<.001
**Placebo/Baseline**						
Right anterior cingulate, BA 32	21	6	44	4	16.96	<.001
Left medial frontal gyrus, BA 6	114	0	−8	48	14.56	<.001
Right medial frontal gyrus, BA 10	36	2	48	14	11.41	<.001
Left precuneus, BA 7	364	0	44	44	20.44	<.001
**Placebo/Week 8**						
Right cingulate gyrus, BA 31	525	6	40	32	28.00	<.001
